# Editorial: Brain Tumors and Neuroinflammation

**DOI:** 10.3389/fncel.2022.941263

**Published:** 2022-06-01

**Authors:** Myriam Catalano

**Affiliations:** Department of Physiology and Pharmacology, Sapienza University of Rome, Rome, Italy

**Keywords:** gliomas, GBM, phenotypic plasticity, tumor microenvironment, neuroinflammation, tumor-associated macrophages, infiltration

Neuroinflammation is the inflammatory response of the brain or the spinal cord like all inflammatory states, characterized by the release of various cytokines, chemokines, reactive oxygen species, and second messengers which, in the nervous system, are produced by glia cells (such as microglia and astrocytes), endothelial cells and peripheral immune cells. It occurs in conditions of altered brain homeostasis, such as in presence of brain tumors, that modify the brain parenchyma and the surrounding microenvironment.

In fact, all nearby cells react to the presence of tumor cells, by modifying their morphology, the expression of membrane receptors and their secretome composition, in response to tumor-released molecules, but also to contact with tumor cells.

Among the primary malignant adult brain cancers, there are low-grade tumors as astrocytomas and oligodendrogliomas and high-grade tumors, as glioblastoma multiforme (GBM). GBM is the most aggressive subtype of malignant brain tumor, with a 14-month median overall survival. Both histological (i.e., cellularity, mitotic figures, necrosis, vascularity) and genetic features (as DNA methylation) profile the classification of brain tumors. These changes can be caused by the malfunctioning of the inflammation process, which can be a new and excellent target for limiting the growth and the aggressiveness of the GBM.

One of the protumoral histological changing is the epithelial-to-mesenchymal cell transition. The Research Topic hosted a work regarding this aspect by Wang et al. on the ubiquitination of the tumor oncogene sphingosine kinase 2 (SphK2) by the neural precursor cell-expressed developmentally downregulated 4-like (NEDD4L) protein. Sphk2 is upregulated in glioma tissues and enhances glioma cell epithelial-to-mesenchymal transition, through the AKT/β-catenin pathway and its ubiquitination suppress glioma cell viability and invasion and promotes apoptosis. This study provides an attractive target for the treatment of glioma.

Among the genetic alterations for gliomas there are the codeletion of chromosome 1p/19q and the mutation of IDH (isocitrate dehydrogenase). The paper by Lv et al. demonstrates that on chromosome 1p or 19q there exist 127 immune-related genes belonging to different classes such as genes coding for cytokines and chemokines, genes that regulate immune cell infiltration and immune checkpoint genes in tumors. All these genes are closely related to the immunosuppressive microenvironment in gliomas and may represent targets for immunotherapeutic strategies.

Another alteration of glioma cells concerns nuclei, that show typical cancerous characteristics, such as grooves, long clefts, and indentations. In the paper by Chung et al. authors demonstrate that nuclear interleukin (IL)-33 plays a key role in glioma progression; it is involved in the chromatin organization and gene positioning, leads to tumor cell transformation and malignancy and induces resistance to the anti-tumor alkylating drug temozolomide. The study provides evidence of this specific nuclear cytokine as a key element in the pathogenesis of glioma-related neuroinflammation.

The topic hosts an interesting review by Mitchell et al. that unravels a very important question regarding neuroinflammation, that is, how excessive activation of the neuro-immune axis leads to primary neuroinflammatory diseases such as multiple sclerosis and antibody-induced encephalitis and how suppression of the same axis promotes birth and growth of primary brain tumors. The review also summarizes the therapeutic attempts for CNS pathologies that target the neuro-immune axis and underlines that GBM is one of the pathologies for which this therapy is extensively studied. The authors give particular attention to the mechanisms that have led to the understanding of the interactions between the GBM microenvironment and the neuro-immune axis that have allowed the development of successful therapeutic strategies for the treatment of this tumor.

Another review hosted in the Topic by Otazu et al. underlines one of the possible targets on the neuro-immune axis for the treatment of GBM which concerns receptors widely expressed by tumor associated macrophages/microglia (TAMs) such as the receptors for advanced glycation end-products (RAGE). RAGE bind to their ligands, such as damage-associated molecular pattern molecules (DAMPs) and to DNA to activate a cascade of signals that lead to overproduction of cytokines, chemokines, adhesion molecules and other similar molecules involved in cellular functions important for tumorigenesis processes such as proliferation, angiogenesis, invasion and migration. The review examines many studies both *in vitro* and *in vivo* in which the antitumor effect of the inhibition of RAGE signaling is reported. These studies have shown that the blocking of the intracellular signals induced by RAGE activation reduces the neuroinflammation status in the case of GBM and emphasizes this immunotherapeutic target in the treatment of this neoplasia.

The reason why this Topic Research is worth reading is that it focuses on the neuroinflammation processa in brain tumors and specifies some of the ways in which it cooperates in tumor progression. Specifically, the Topic collected important results in which it has been identified a biochemical process (ubiquitination), a genetic mutation (a codeletion) and a specifical nuclear localization as key regulators of brain tumor-related neuroinflammation. All of them represent potential therapeutic targets in the treatment of this devastating pathology. The reviews collected in the Topic summarized the relationship between glioblastoma microenvironment and the neuro-immune axis, and have highlighted how important the neuroimmune axis is in regarding its therapeutic use. In conclusion, this Topic Research has collected important milestones with respect to the possible use of neuroinflammation processes as a target for therapies aimed at limiting the progression of GBM, expressing a collection useful to all researchers of the most widespread primary brain tumor in adults.

## Author Contributions

MC analyzed the articles published in the topic and reported what was the contribution of each to the topic.

## Conflict of Interest

The author declares that the research was conducted in the absence of any commercial or financial relationships that could be construed as a potential conflict of interest.

## Publisher's Note

All claims expressed in this article are solely those of the authors and do not necessarily represent those of their affiliated organizations, or those of the publisher, the editors and the reviewers. Any product that may be evaluated in this article, or claim that may be made by its manufacturer, is not guaranteed or endorsed by the publisher.

